# Significant differences in alkaloid content of *Coptis chinensis *(*Huanglian*), from its related American species

**DOI:** 10.1186/1749-8546-4-17

**Published:** 2009-08-24

**Authors:** Shreya Kamath, Matthew Skeels, Aswini Pai

**Affiliations:** 1 Department of Chemistry, St Lawrence University, Canton, New York 13617, USA; 2 Department of Biology, St Lawrence University, Canton, New York 13617, USA

## Abstract

**Background:**

The growing popularity of Chinese herbal medicine in the United States has prompted large-scale import of raw herbs from Asia. Many of the Asian herbs have phylogenetically related North American species. We compared three phylogenetically related species, namely *Coptis chinensis *(*Huanglian*), *Hydrastis canadensis *and *Coptis trifolia *to show whether they can be substituted by one another in terms of alkaloid content.

**Methods:**

We used microwave assisted extraction to obtain alkaloids berberine, coptisine, palmatine and hydrastine. High performance liquid chromatography (HPLC) was used to quantify each alkaloid.

**Results:**

*Hydrastis canadensis *has the most berberine, whereas *Coptis trifolia *has the most coptisine. Hydrastine and palmatine were unique to *Hydrastis canadensis *and *Coptis chinensis *respectively.

**Conclusion:**

Neither *Hydrastis canadensis *nor *Coptis trifolia *contains all the alkaloids found in *Coptis chinensis *used in Chinese medicine. Substitutes of this Chinese species by its American relatives are not recommended.

## Background

Phylogenetically related plant species exhibit phytochemical similarities [1]. For example, taxol is an anti-cancer chemical compound initially isolated from the bark of a rare tree species *Taxus brevifolia *(Pacific Yew tree); however, a closely related and more available species, *Taxus cuspidata *was later proved to be a more commercially viable source for taxol [2]. The growing popularity of Chinese herbal medicine in the United States has prompted large-scale import of raw herbs from Asia [3]. As many Chinese medicinal plants are evolutionarily related to their North American congeners, the North American species may be possible phytochemical substitutes to the Chinese medicinal herbs [4]. For example, North American ginseng (*Panax quinquifolium*) has become the commercial alternative to the rarer Chinese ginseng (*Panax ginseng*) [5]. Growing native substitutes can potentially promote local industry and reduce the ecological risks of cultivating exotic species; however, there is little information available on possible North American phytochemical substitutes to Chinese medicinal herbs.

The present study investigates the alkaloid content in *Coptis trifolia *(American goldthread) and *Hydrastis canadensis *(goldenseal), two herbs found in the United States, and *Coptis chinensis *(*Huanglian*, Chinese goldthread), a Chinese medicinal herb imported from China. The three species belong to the same Ranunculaceae family and have alkaloid rich rhizomes. Phylogenetically related to *Hydrastis canadensis*, the genus *Coptis *has several North American and Asian species [6].

During the 18^th ^century, Native Americans used the rhizome of *Coptis trifolia *to treat mouth sores, poor digestion and infections. The species had become scarce due to over harvesting and/or loss of habitat caused by human activities. It was substituted with *Hydrastis canadensis*, which remains available commercially to this date despite the fact that the growth of *Coptis trifolia *has recovered in the northeastern United States [7]. *Hydrastis canadensis *is an 'at risk' species due to the potential for over harvest but can be cultivated on a small scale [8]. Both species are phylogenetically related to *Coptis chinensis *which is used to treat digestive and respiratory disorders in Chinese medicine [9].

A review of available literature indicates that there has been considerably more pharmacological research on *Coptis chinensis *than *Hydrastis canadensis *and *Coptis trifolia*. Current findings show some of their similarities. Bioassays on *Coptis chinensis *and *Hydrastis canadensis *indicate that both herbs possess anti-microbial activities [10,11]. In addition, *Coptis chinensis *is anti-inflammatory [12] and *Hydrastis canadensis *may improve immune function [13]. Pharmacological studies on *Coptis trifolia *are still lacking. *Coptis chinensis *contains alkaloids such as berberine, palmatine, epiberberine, coptisine, jaterorhizine and columbamine. *Hydrastis canadensis *also contains alkaloids such as berberine, hydrastine, hydrastinine and canadine [4,14]. Isoquiniline alkaloids in *Coptis trifolia *were suggested to be similar to those in *Coptis chinensis *yet they have not been fully characterized [3]. Berberine, a benzylisoquinoline alkaloid, has been found in all three species and has been demonstrated to be anti-carcinogenic and anti-microbial [15].

The present study aims to investigate whether berberine, coptisine, hydrastine and palmatine (major isoquinoline alkaloids) are quantitatively comparable in content among rhizomes of *Hydrastis canadensis, Coptis trifolia *and *Coptis chinensis*.

## Methods

*Hydrastis canadensis *(*n *= 20) was obtained from Horizon Herbs, Oregon, USA. *Coptis trifolia *(*n *= 20) was harvested from the Wachmeister field station northern New York, USA. *Coptis chinensis *rhizomes (*n *= 20) were obtained from a supplier in Montreal, Canada. The whole plants of *Hydrastis canadensis *and *Coptis trifolia *were authenticated by comparing their morphology with type specimens at the St Lawrence University herbarium [16]. The *Coptis chinensis *rhizomes were authenticated by comparing the high performance liquid chromatography (HPLC) peaks to those from published data [17,18].

Acetonitrile and methanol (HPLC-grade) were obtained from Pharmco-AAPER (USA). Hexane and phosphoric acid (ACS grade) were obtained from JT Baker (USA). Berberine, palmatine and coptisine were obtained from Sigma-Aldrich (USA) and hydrastine was purchased from MP Biomedical (USA).

All rhizomes of the three herbs were dried at 75°C in a forced air oven for 72 hours and subsequently were ground into fine powder with a mortar and pestle. Alkaloids were extracted with a microwave assisted extraction (MAE) technique [19] and quantified with HPLC [20]. Samples were soaked in 30 mL of extraction solvent. Solvents described by Anderson *et al. *[21] and Li *et al. *[22] were used. Specifically, an extraction solvent of 50:50:0.1 (v/v) hexane-ethanol-phosphoric acid mixture was used for *Hydrastis canadensis *while a 100:0.1 (v/v) methanol-phosphoric acid was used for *Coptis trifolia *and *Coptis chinensis*. These acidified polar solvents effectively extract the isoquinoline alkaloids in their highly soluble, protonated forms. We carried out the method of Li *et al. *[22], except that MAE was employed to shorten the overnight extraction time. The samples were placed in a Aurora MW500 microwave (Aurora Instruments, Canada) programmed to ramp temperature from 25°C to 120°C in four minutes, followed by 90 minutes of extraction at 120°C. Filtered extracts were dried in a Heidolph roto-vaporizer (Heidolph Elektro, Germany) under vacuum of 367 mbar at 70°C and 150 rpm. The dried residue was dissolved in a minimal volume of 100% HPLC-grade acetonitrile, ranging from 400 μl to 2 mL (depending on the residue quantity) for 24 hours.

HPLC analyses were performed according to the validated method of Weber *et al. *[20] on a ThermoSeparations SpectraSYSTEM (Thermo Scientific, USA) chromatograph with an Agilent Eclipse XDB-C18 (4.6 mm × 250 mm × 5 μm) column (Agilent Technologies, USA). The flow rate of the mobile phase, which consisted of 0.2 M ammonium acetate (pH 4.85) and acetonitrile (69:31, v/v), was maintained at 1.0 mL per minute [20] and absorbance was measured at 235 nm and 266 nm. Alkaloids in each sample were identified according to their standard retention times and characteristic A_235_/A_266 _ratios. To corroborate alkaloid identity in samples, we used 'spiking tests' with known standards. Peak area at 266 nm was used to quantify alkaloid content.

Stock solutions were prepared by weighing 5 mg of standard with a Cahn C-35 microbalance (Themo Scientific, USA) into individual 10 mL volumetric flasks and diluting it to volume with water and acetonitrile (10:90 v/v). A 5 mL aliquot of each stock solution was transferred into a 25 mL volumetric flask and diluted to volume with water and acetonitrile (70:30, v/v). Wrapped in aluminum foil and refrigerated, the stock solutions were stable for over two months. Various volumes of standards were analyzed in triplicates to produce standard curves (0.5 to 2 μg). Individual calibration curves, plotted for each alkaloid, were linear (R^2 ^> 0.99) over the range of interest.

As the data did not meet the assumption of equal variance, we used non-parametric tests instead of parametric tests for statistical analyses. Kruskal-Wallis test was used to compare Berberine content was compared amongbetween *Hydrastis canadensis*, *Coptis trifolia *and *Coptis chinensis*, whereas Mann-Whitney U test was used to compare coptisine content between *Coptis trifolia *and *Coptis chinensis*.

## Results and discussion

**Figure 1:**
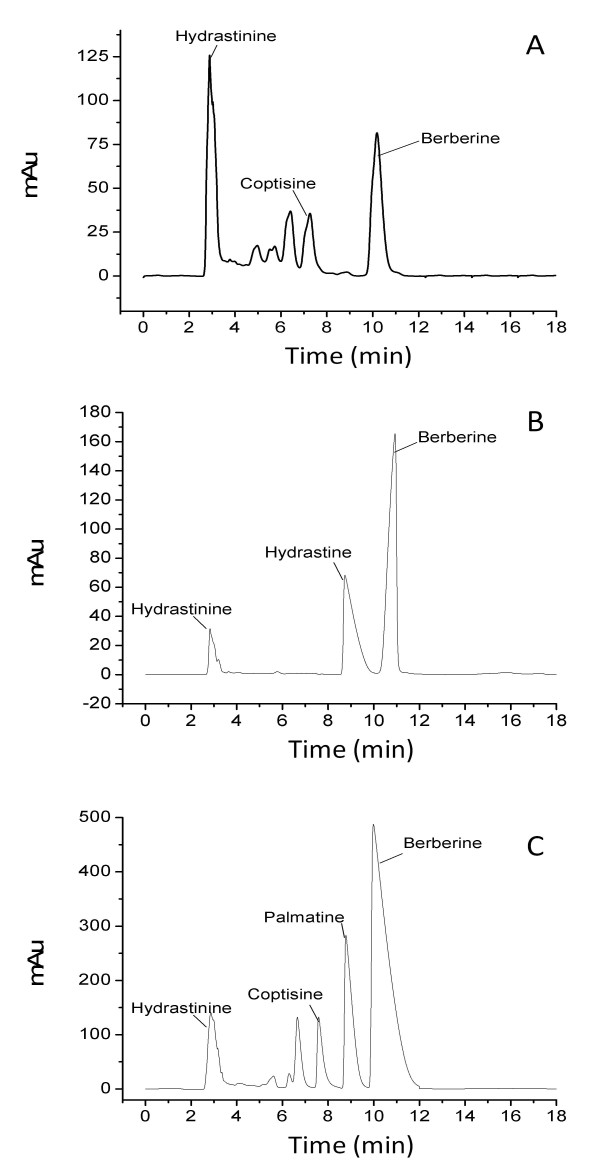
**Representative HPLC chromatograms**. (A) *Coptis trifolia. *(B) *Hydrastis canadensis. *(C) *Coptis chinenesis. *All three herbs contain hydrastinine and berberine. Coptisine is present in *Coptis trifolia *and *Coptis chinenesis. *Palmatine is only detected in *Coptis chinensis*, whereas hydrastine is only present in *Hydrastis canadensis.*

Figure 1 shows the representative chromatograms of the three herbs. Berberine was quantified in all three species. Coptisine was quantified in *Coptis chinensis *and *Coptis trifolia*. Hydrastine and palmatine were unique to *Hydrastis canadensis *and *Coptis chinensis *respectively but absent in *Coptis trifolia*. The Kruskal-Wallis test indicates a significant difference (H = 49.67, *P *< 0.0001) in berberine content among the three species. *Hydrastis canadensis *has the greatest berberine content, followed by *Coptis chinensis *and *Coptis trifolia *(Table 1). The Mann-Whitney U test indicates a significant difference (Z = 3.45, *P *< 0.0001) in the coptisine content in the rhizomes of *Coptis trifolia *and *Coptis chinensis*. *Coptis trifolia *has higher mean coptisine content than that of *Coptis chinensis*.

**Table 1:** Alkaloid content in rhizomes (*n *= 20) of *Hydrastis canadensis, Coptis chinensis *and *Coptis trifolia *(Similar alkaloids differ significantly among species at *P *< 0.0001.)

**Species**	**Major alkaloids**	**Mean (SD) alkaloid content (mg/g of rhizome)**
*Hydrastis canadensis*	Berberine	16.76 (3.42)
	Hydrastine	4.81 (1.48)
*Coptis chinensis*	Berberine	10.60 (1.36)
	Coptisine	2.35 (0.27)
	Palmatine	2.91 (0.38)
*Coptis trifolia*	Berberine	3.80 (1.90)
	Coptisine	3.44 (1.45)

These results show that *Hydrastis canadensis *is the best source for berberine, whereas *Coptis trifolia *is the best source for coptisine. Neither of the two North American species has all the alkaloids found in their Chinese congener *Coptis chinensis*. *Hydrastis canadensis *and *Coptis trifolia *together contain all the alkaloids found in *Coptis chinensis *except palmatine. Therefore, a combined formulation of the two North American herbs may provide most of the alkaloids in *Coptis chinensis *and may be acceptable to the North American dietary supplements industry if only a few key phytochemicals are required [23]. As growth conditions can influence alkaloid content of a plant, further investigation is indeed needed on how cultivation of the American species may make the substitution possible.

If there is any difference in photochemical content, possible interactions [24], toxicity of the active constituents and the phytochemicals introduced from the American species should be studied. Otherwise, substitution of the Chinese species by the American ones is not recommended.

## Conclusion

Neither *Hydrastis canadensis *nor *Coptis trifolia *contains all the alkaloids found in *Coptis chinensis *used in Chinese medicine. Substitutes of this Chinese species by its American relatives are not recommended.

## Abbreviations

MAE: microwave assisted extraction; HPLC: high performance liquid chromatography; SD: standard deviation

## Competing interests

The authors declare that they have no competing interests.

## Authors' contributions

AP conceived the study design and performed the statistical analyses. MS conceptualized and supervised the HPLC. SK performed the HPLC. All authors participated in the writing of the manuscript. All authors read and approved the final version of the manuscript.
